# Medical Students' Voluntary Service During the COVID-19 Pandemic in Poland

**DOI:** 10.3389/fpubh.2021.618608

**Published:** 2021-04-13

**Authors:** Jan Domaradzki, Dariusz Walkowiak

**Affiliations:** ^1^Department of Social Sciences and Humanities, Poznan University of Medical Sciences, Poznań, Poland; ^2^Department of Organization and Management in Health Care, Poznan University of Medical Sciences, Poznań, Poland

**Keywords:** COVID-19, pandemic (COVID-19), students, voluntary service, future healthcare professionals

## Abstract

From the very first moment coronavirus struck, medical students volunteered to support healthcare professionals' fight against the COVID-19 pandemic. To learn more about future healthcare professionals' volunteering during such an outbreak, we conducted a survey among 417 students of Poznan University of Medical Sciences. Our findings suggest that although numerous studies demonstrate that traditional, value-based volunteering is decreasing, and especially higher education students are more oriented toward their own career, in the times of the current health crisis, young peoples' involvement in volunteering has been mainly driven by altruism and the ethical imperative to serve their community, their fellow healthcare professionals and their patients. Thus, while the prime role of the volunteering was to relieve the healthcare system, it also reinforced such important medical values as altruism, public service and professional solidarity. Moreover, it proved that whilst risk is inherent to medicine, the students' volunteering is truly a moral enterprise.

## Introduction

Ever since the first case of severe acute respiratory syndrome coronavirus 2 (SARS-CoV-2) infection in Poland was confirmed on 4 March 2020, the Polish government has imposed different types of lockdown-type control measures, including the closing of universities, which moved to online lectures, and on 20 March a state of epidemic was announced. However, although medical students were pulled off from the hospital's medical universities in the country, many universities started encouraging future healthcare professionals to volunteer. Consequently, although they faced concerns about their health and education, thousands of Polish students from the faculties of Medicine, Pharmacy and Health Sciences have supported the fight against the coronavirus pandemic and engaged in voluntary service in local hospitals, sanitary*-*epidemiological stations, emergency units, hospital pharmacies, the university's diagnostic laboratory and local call centers, and soon most places were filled by volunteers and many other students are still waiting for their turn.

This is of key importance, because in many countries, i.e., Italy or Spain, the healthcare systems reached a breaking point and have been seriously burdened by the COVID-19 pandemic and the struggle with insufficient medical personnel. Moreover, while the media has publish many distressing images of ill and dead people in various European countries, Poland has difficulty in retaining its health professionals and has the lowest number of physicians per 100,000 inhabitants in the European Union, and the number of practicing nurses in the country is also one of the lowest in the EU (1).

Moreover, while until October 14, 141 804 cases of infections were reported in Poland, 3 217 patients died and 83 847 recovered (2), it is healthcare professionals that are at the increased risk of being infected as 17% of those infected are health professionals; near 4,000 medics were infected (including 986 physicians, 2 393 nurses, 212 midwifes, 89 dentists, 75 laboratory diagnosticians, 68 paramedics and 64 pharmacists), 31 077 were quarantined (8 881 physicians, 18 495 nurses, 1 644 midwifes, 824 dentists, 674 pharmacists, 451 laboratory diagnosticians and 108 paramedics), 678 were hospitalized (398 nurses, 194 physicians, 31 paramedics, 27 midwifes, 12 dentists, eight pharmacists and five laboratory diagnosticians) and 13 have died (seven physicians, six nurses) (3). Thus, although some countries asked medical students to step down, this is not the first time when future health professionals serve at the frontline of the battle with the pandemic (4, 5). Not surprisingly, medical, nursing, midwifery, physiotherapy and pharmacy students have adapted to many new roles and help in administrative and office work, in emergency rooms and hospital wards, interview patients, care for outpatients through telemedicine, translate English texts about COVID-19, help with making supplies of personal protective equipment, sew protective masks or provide child care for healthcare workers.

At the same time, while some theories concerning volunteering focus on personal motives and emphasize rational action and a cost-benefit analysis, others stress the role of accessible social resources such as organizational activity and social ties (6, 7). However, researchers have also investigated the contextual effects on volunteering and have paid attention to the impact of organizational, community and regional characteristics on individual decisions to volunteer (8, 9). Moreover, it is often argued that volunteerism has much in common with social activism and that both types of collective engagement are not so much initiated by the state or by political professionals but by collectives who act together for a common and specific purpose (10, 11). Indeed, in response to the crisis situation caused by the COVID-19 outbreak, Poznan University of Medical Sciences (PUMS), in collaboration with university's student organizations, initiated a COVID-19 student volunteering project. And while students who joined the project were offered various compensations, including credits for a compulsory internship or flexible assessment of e-learning outcomes, the project itself emphasized that nurturance and care for others were deeply embedded in the role of health professionals. Nevertheless, many scholars argue that the nature of volunteering is being restructured and that volunteers' motivations are changing as the old or traditional forms of volunteering (long-term, based on membership, inspired by altruistic values and the importance of social interactions and connected to religious or political communities) are being replaced by the modern type of volunteering (project-oriented, based on career development and personal growth and not rooted in a local community) (12–16).

Thus, while some studies have described the knowledge and attitudes toward the COVID-19 among medical students (17, 18), this study focuses on students' experience of the pandemic and describes their experience with voluntary service during the COVID-19 pandemic. It also analyses the reasons behind the future healthcare professionals' involvement in voluntary service during the COVID-19 outbreak in Poland.

## Materials and Methods

The study was conducted between 5 of May and 30 of June 2020. Participants were students enrolled in different faculties of Poznan University of Medical Sciences, Poland. An online questionnaire which was posted on an online platform was used. The process of elaborating the questionnaire followed the guidelines of the European Statistical System (19). The questionnaire consisted of four main sections: students' experience of the pandemic, students' experience with voluntary service during the COVID-19 outbreak, the reasons students' became involved in voluntary service, and socio-demographics. It was reviewed by a panel of experts and revised based on their comments. The final version of the questionnaire was approved by the University Student Council Board (USCB). All the participants received an invitation letter and informed consent was obtained from all individuals included in the study. The results are presented as descriptive statistics.

## Results

From the beginning of the pandemic, PUMS received applications from students who wished to support hospitals and other units of the healthcare system with their work. By the end of May, 741 of them had started volunteering. They were directed to help both university units and those under the control of the local authorities. Of this group, 417 students (56.3%) completed the questionnaire. Our group consisted of 301 females and 116 males (Table 1), representing all degree courses and years of study, but most of them, 256 (61.4%), were students of the medical faculty, which is the most numerous. The majority of volunteers 300 (71.9%) had various types of volunteer experience before the pandemic, and 72 (17.3%) had been volunteers more than 10 times.

**Table 1:** Socio-demographic characteristics of students.

**Characteristics**	***N* (%)**
**Gender**	
Female	301 (72.2)
Male	116 (27.8)
**Year of study**	
1	40 (9.6)
2	87 (20.9)
3	56 (13.4)
4	99 (23.7)
5	74 (17.7)
6	61 (14.6)
**Faculty**	
Medicine	256 (61.4)
Nursing	42 (10.1)
Pharmacy	23 (5.5)
Electroradiology	20 (4.8)
Medical analytics	19 (4.5)
Dentistry	14 (3.4)
Midwifery	11 (2.6)
Medical rescue	10 (2.4)
Other	22 (5.3)
**How many times have you volunteered before?**	
0	117 (28.1)
1	26 (6.2)
2	62 (14.9)
3–5	106 (25.4)
6–10	34 (8.1)
>10	72 (17.3)

The main feelings evoked by the coronavirus outbreak in our respondents were fear over their loved ones (65.5%) and the willingness to act (59.7%) (Table 2). Simultaneously, the vast majority of students (87%) believed that the control measures imposed by the authorities were justified. Before deciding to participate in volunteering, 59.2% of students consulted their parents and 51.3% their university friends.

**Table 2:** Students' experience of a pandemic.

	***N* (%)**
What were your feelings after hearing about the coronavirus outbreak?	
Fear for loved ones	273 (65.5)
Willingness to act	249 (59.7)
Fear about my own future	116 (27.6)
Anger	153 (36.7)
Nothing, it was irrelevant to me	24 (5.8)
Other	28 (6.7)
What were your feelings after hearing about the control measures ruled by the government?	
That the government's reaction was right	363 (87)
That the government is overreacting	45 (10.8)
It was irrelevant to me	9 (2.2)
Did you consult your decision on engaging into voluntary service with anybody?	
Parents	247 (59.2)
Siblings	61 (14.6)
Partner	175 (42)
My fellow students	214 (51.3)
My university teacher	18 (4.3)
A priest	3 (0.7)
No	72 (17.2)

The largest group of students participated in administrative work (39.8%), helped in emergency rooms (33.3%), took the medical history of the patients (21.3%) and helped with medical procedures in a hospital ward (18%) (Table 3). Others helped with making supplies of personal protective equipment and gave telephone advice in a sanitary*-*epidemiological station (7%). At the same time, most volunteers were not so much concerned over the possibility of being infected (31.4%) as they were about how the pandemic might affect the situation in the country (47.7%) and their studies (47.5%). Additionally, almost one third of students were afraid that due to the pandemic the healthcare system might be seriously burdened or even collapse (31.7%) and that the pandemic might affect their economic situation (27.3%). Interestingly, while 16.5% of students worried that they may not handle the voluntary service, 14.4% had no worries.

**Table 3:** Students' experience with voluntary service during the COVID-19 pandemic.

	***N* (%)**
What do you do during voluntary service?	
I help in administrative and office work	166 (39.8)
I help with the documentation of patients and persons under epidemiological surveillance	49 (11.8)
I give telephone advice in a sanitary*-*epidemiological station	29 (7)
I take medical history from those infected	89 (21.3)
I give medical advice on the Internet and at a telephone information desk	20 (4.8)
I help in the emergency room	139 (33.3)
I help with medical procedures in a hospital ward	75 (18)
I help in the university's diagnostic laboratory	24 (5.8)
I sew protective masks	14 (3.4)
I help with making supplies of personal protective equipment	35 (8.4)
I help those in need, i.e., the seniors, the children	21 (5)
I help with the translation of English texts about COVID-19	16 (3.8)
Were you anxious about anything during your voluntary service?	
That I can get infected	131 (31.4)
That the healthcare system may collapse	132 (31.7)
That the pandemic will affect my studies	198 (47.5)
That pandemic will affect the situation in the country	199 (47.7)
That I will not handle it	69 (16.5)
That the pandemic will affect my economic situation	114 (27.3)
I had no worries	60 (14.4)
What was people's, including your colleagues', reaction to your voluntary service?	
Positive	365 (87.5)
Indifferent	31 (7.5)
Negative	21 (5)
Does voluntary service meet your expectations?	
Yes	329 (78.9)
No	88 (21.1)
Do you regret your decision to join the voluntary service?	
Yes	15 (3.6)
No	402 (96.4)
Do you find voluntary service harder than you expected?	
Yes	61 (14.6)
No	356 (85.4)

More than 87% of volunteers declared having met with positive reactions either form their families, fellow colleagues or friends. Moreover, while the majority declared that the voluntary service met their expectations (78.9%), very few regretted their decision to join the COVID-19 student volunteering project (3.6%). And although some students admitted being concerned over their qualifications which might be inadequate to their responsibilities, over 85% of students declared that voluntary service was not as hard as than they had expected.

For 23.5% of the students the most important reason to engage in voluntary service during the pandemic was their belief that the role of medics is to engage and help regardless of the risk, while 20.9% believed that it is important to help others, 12.9% wanted to be a part of something important and 12.7% wanted to gain experience needed in their future profession (Table 4).

**Table 4:** Reasons of students' involvement in voluntary service during the COVID-19 pandemic.

**What was the main reason to engage in voluntary service during the COVID-19 pandemic**	***N* (%)**
To put my voluntary participation into my future application documents	8 (1.9)
To gain experience needed in my future profession	53 (12.7)
To establish new connections that will be useful in the future	2 (0.5)
I believe it is important to help others	87 (20.9)
I believe that the role of medics is to engage and help whatever the risk	98 (23.5)
It gives me the opportunity to pay back for all I have received myself	5 (1.2)
I wanted to be a part of something important	54 (12.9)
To experience the adventure	9 (2.1)
It gives me the opportunity to realize my passion	11 (2.6)
It is better than sitting at home and studying, or to be bored	41 (9.8)
To meet new people, make new connections and friends	4 (1)
I was advised by my teacher/parent that I may benefit from it	4 (1)
I was encouraged by a friend who also volunteered	5 (1.2)
Completion of work placement	22 (5.3)
Other	14 (3.4)

Volunteers were also asked to rate on a scale from 1 (not significant) to 5 (very important) various reasons for volunteering (Table 5). Fifty eight point eight percentge of the students gave the highest rating to the option “help others,” and 46.5% chose “giving something from myself to the community.” On the other hand, 4.1% wanted to enhance their professional résumé.

**Table 5:** Students' motivations.

	**1**	**2**	**3**	**4**	**5**	**Mean**
To enhance my professional résumé	180	85	80	55	17	2.15
To get new knowledge and skills	34	41	90	109	143	3.69
To gain professional experience	40	48	79	130	120	3.58
To make new contacts that might help me in the future	76	98	115	96	32	2.78
To help others	8	12	39	113	245	4.38
To give something from myself to the community	15	26	57	125	194	4.1
To realize the duty of public service inherent to the medical profession	60	51	78	96	132	3.45
To help succeed in the fight against the pandemic	34	41	82	145	115	3.64
To participate in something important	39	40	85	115	138	3.66
To have a sense of duty and pride	48	61	76	120	112	3.45
To realize my passion	36	38	105	124	114	3.58
To experience the adventure and to tell my future kids that I was a part of it	110	85	89	83	50	2.71
To fill free time	106	63	85	97	66	2.89
To make new friends and establish new connections	103	99	108	72	35	2.61
To work with other people	39	61	92	126	99	3.44
To gain the recognition of my professors, family and friends	196	113	64	37	7	1.91

## Discussion

Despite the closure of all medical universities in Poland, hundreds of future healthcare professionals volunteered in their communities and local hospitals to provide medical assistance and guidance to the public. Although students were aware that they were not full-fledged members of the medical teams, that their ability to provide care was limited and that their primary role was to learn medicine, most volunteers believed that it is their duty to serve society, help medical professionals and care for patients. Moreover, even though respondents felt anxious about the social, economic and health disruptions caused by the virus, their future and of the possibility of being infected, they eagerly made a commitment and took the Hippocratic Oath to care for those in need very seriously.

Thus, while some research suggests that among higher education students a new type of the so called résumé building volunteering becomes more popular (12–14), our study shows that in the times of the health crisis caused by the COVID-19 outbreak, young peoples' involvement in voluntary service is mainly driven by altruism and public service, and can be described as traditional, value-based volunteering. Even though some students stepped up for more individualistic and career reasons, i.e., they hoped that their voluntary service will help them to gain new knowledge and skills, develop their personal career or allow them to pass their summer internships, many others did so to fulfill the calling that the healthcare profession entails. This supports the observation made by Gage and Thapa (15) who argue that students' volunteerism is mostly driven by their desire to help others and expand their character. Thus, it seems that while for many students résumé building and gaining new knowledge and skills was somehow important, it was rather an additional benefit and not a prime motivation (16). On the contrary, as most students were more driven by the ethical imperative to serve their community, healthcare professionals and their patients, they stepped forward out of a sense of civic responsibility, believed that the healthcare service is a unique vocation and that as future health professionals it was their duty to engage and help, whatever the risk (4, 5).

## Conclusion

While the prime role of students' voluntary service during the COVID-19 pandemic was to relieve the healthcare system before it reaches a personnel crisis similar to that in other countries, it also helped students to learn new practical skills, rethink ethical dilemmas learnt during their courses and, most importantly, reinforced such important values of medical ethos as: altruism, public service and (professional) solidarity. Moreover, by undertaking a variety of tasks, from administrative and office work, giving telephone advices in call centers, helping in hospital wards and university's diagnostic laboratories to the translating of English texts about COVID-19 and sewing protective masks, students have proved that although risk to life is inherent to the healthcare service, medicine is truly a moral enterprise. Finally, this study shows that student's voluntary service during the coronavirus pandemic is an important part of service learning (20) which should become an integral component of medical education.

### Strengths and Limitations

Of course, our study does have its limitations. First, as we analyzed responses from students from only one medical university in the country, the study has a local dimension. Consequently, it would be desirable to compare the findings from other medical universities. However, to the best of our knowledge, no research on students' volunteering during the COVID-19 pandemics at other Polish universities has been done. Moreover, we believe that because this is a pilot study, it may stimulate further research on students' voluntary work during the COVID-19 pandemic. Second, although the response rate was moderately high, the results represent only the opinions of students who agreed to participate in the study and cannot be generalized for the entire student population either in Poznan or in Poland. Third, non-random sampling is another limitation as it prevented an analysis of the socio-demographic, structural and socio-cultural background of the issues discussed in our research. Finally, as this study is based on the quantitative method only, to understand better students' motivations, opinions and lived experiences, further in-depth studies using qualitative methods would be required. Nevertheless, we believe that as this is the first study on students' voluntary service during a coronavirus pandemic in Poland, it may stimulate further research on the topic.

## Data Availability Statement

The raw data supporting the conclusions of this article will be made available by the authors, without undue reservation.

## Ethics Statement

Ethical review and approval was not required for the study on human participants in accordance with the local legislation and institutional requirements. The patients/participants provided their written informed consent to participate in this study.

## Author Contributions

JD designed the study, collected the data, and wrote the original draft of the manuscript. DW performed the statistical analyses. JD and DW conducted the literature search and analyses, discussed the results and interpreted the data. JD and DW edited and approved the final version of the manuscript.

## Conflict of Interest

The authors declare that the research was conducted in the absence of any commercial or financial relationships that could be construed as a potential conflict of interest.

## Abbreviations

COVID-19: Coronavirus Disease 2019
SARS-CoV-2: Severe acute respiratory syndrome coronavirus 2
PUMS: Poznan University of Medical Sciences
USCB: University Student Council Board.
